# Supplementary material for: The effects of genital myiasis on the diversity of the vaginal microbiota in female Bactrian camels
Source: BMC Vet Res. 2022 Mar 5;18:87. doi: 10.1186/s12917-022-03189-5 (PMC8897907; doi:10.1186/s12917-022-03189-5)
Supplement: Supplementary file 5 — Additional file 5. [file 12917_2022_3189_MOESM5_ESM.zip › MPL201709200_16s_yy/Treat1/css/js_css/jsPDF-master.zip!/jsPDF-master/docs/examples_js_editor.js.html]

examples/js/editor.js - Documentation


## Home

### Classes

- FontObject
- jsPDF
- PubSub

### Global

- addFont
- addMetadata
- addPage
- CapJoinStyles
- circle
- CssColors
- ellipse
- getFontList
- html2pdf
- init
- jsPDFEditor
- lines
- output
- pdf\_test\_harness\_init
- rect
- requirejs
- reset
- roundedRect
- save
- setDrawColor
- setFillColor
- setFont
- setFontSize
- setFontStyle
- setLineCap
- setLineJoin
- setLineWidth
- setProperties
- setTextColor
- text
- toUrl
- triangle
- triggerEvent
- update

# examples/js/editor.js

```
/**
 * jsPDFEditor
 * @return {[type]} [description]
 */
var jsPDFEditor = function() {

	var editor,demos = {
		'images.js': 'Images',
		'font-faces.js': 'Font faces, text alignment and rotation',
		'from-html.js': 'HTML Renderer (Early stages)',
		'two-page.js': 'Two page Hello World',
		'circles.js': 'Circles',
		'font-size.js': 'Font sizes',
		//'kitchen-sink.js': 'Kitchen Sink', // @TODO
		'landscape.js': 'Landscape',
		'lines.js': 'Lines',
		'rectangles.js': 'Rectangles',
		'string-splitting.js': 'String Splitting',
		'text-colors.js': 'Text colors',
		'triangles.js': 'Triangles',
		'user-input.js': 'User input',
		//'html2canvas.js': '** NEW: addHTML()',
		'acroForm.js': 'AcroForms',
		'autoprint.js': 'Auto print'
	};

	var aceEditor = function() {
		editor = ace.edit("editor");
		// editor.setTheme("ace/theme/twilight");
		//editor.setTheme("ace/theme/ambiance");
		editor.setTheme("ace/theme/github");
		editor.getSession().setMode("ace/mode/javascript");
		editor.getSession().setUseWorker(false); // prevent "SecurityError: DOM Exception 18"

		var timeout;
		editor.getSession().on('change', function() {
			// Hacky workaround to disable auto refresh on user input
			if ($('#auto-refresh').is(':checked') && $('#template').val() != 'user-input.js') {
				if(timeout) clearTimeout(timeout);
				timeout = setTimeout(function() {
					jsPDFEditor.update();
				}, 200);
			}
		});
	};

	var populateDropdown = function() {
		var options = '';
		for (var demo in demos) {
			options += '<option value="' + demo + '">' + demos[demo] + '</option>';
		}
		$('#template').html(options).on('change', loadSelectedFile);
	};

	var loadSelectedFile = function() {
		if ($('#template').val() == 'user-input.js') {
			$('.controls .checkbox').hide();
			$('.controls .alert').show();
			jsPDFEditor.update(true);
		} else {
			$('.controls .checkbox').show();
			$('.controls .alert').hide();
		}

		$.get('examples/js/' + $('#template').val(), function(response) {
			editor.setValue(response);
			editor.gotoLine(0);

			// If autorefresh isn't on, then force it when we change examples
			if (! $('#auto-refresh').is(':checked')) {
				jsPDFEditor.update();
			}

		}, 'text').error(function() {

			$('.template-picker').html('<p class="source">More examples in <b>examples/js/</b>. We can\'t load them in automatically because of local filesystem security precautions.</p>');

			// Fallback source code
			var source = "// You'll need to make your image into a Data URL\n";
			source += "// Use http://dataurl.net/#dataurlmaker\n";
			source += "var imgData = '';\n";
			source += "\n";
			source += "var doc = new jsPDF();\n";
			source += "\n";
			source += "doc.setFontSize(40);\n";
			source += "doc.text(40, 30, \"Octocat loves jsPDF\", 4);\n";
			source += "doc.addImage(imgData, 10, 40, 180, 180);\n";
			editor.setValue(source);
			editor.gotoLine(0);
		});
	};

	var initAutoRefresh = function() {
		$('#auto-refresh').on('change', function() {
			if ($('#auto-refresh').is(':checked')) {
				$('.run-code').hide();
				jsPDFEditor.update();
			} else {
				$('.run-code').show();
			}
		});

		$('.run-code').click(function() {
			jsPDFEditor.update();
			return false;
		});
	};

	var initDownloadPDF = function() {
		$('.download-pdf').click(function(){
			eval('try{' + editor.getValue() + '} catch(e) { console.error(e.message,e.stack,e); }');

			var file = demos[$('#template').val()];
			if (file === undefined) {
				file = 'demo';
			}
			if (typeof doc !== 'undefined') {
				doc.save(file + '.pdf');
			} else if (typeof pdf !== 'undefined') {
				setTimeout(function() {
					pdf.save(file + '.pdf');
				}, 2000);
			} else {
				alert('Error 0xE001BADF');
			}
		});
		return false;
	};

	return {
		/**
		 * Start the editor demo
		 * @return {void}
		 */
		init: function() {

			// Init the ACE editor
			aceEditor();

			populateDropdown();
			loadSelectedFile();
			// Do the first update on init
			jsPDFEditor.update();

			initAutoRefresh();

			initDownloadPDF();
		},
		/**
		 * Update the iframe with current PDF.
		 *
		 * @param  {boolean} skipEval If true, will skip evaluation of the code
		 * @return
		 */
		update: function(skipEval) {
			setTimeout(function() {
				if (! skipEval) {
					eval('try{' + editor.getValue() + '} catch(e) { console.error(e.message,e.stack,e); }');
				}
				if (typeof doc !== 'undefined') try {
					if (navigator.msSaveBlob) {
						// var string = doc.output('datauristring');
						string = 'http://microsoft.com/thisdoesnotexists';
						console.error('Sorry, we cannot show live PDFs in MSIE')
					} else {
						var string = doc.output('bloburi');
					}
					$('.preview-pane').attr('src', string);
				} catch(e) {
					alert('Error ' + e);
				}
			}, 0);
		}
	};

}();

$(document).ready(function() {
	jsPDFEditor.init();
});
```

  

Documentation generated by JSDoc 3.4.1 on Mon Oct 03 2016 12:36:18 GMT+0100 (BST) using the docdash theme.
